# Retraction: *Pseudomonas aeruginosa*–induced nociceptor activation increases susceptibility to infection

**DOI:** 10.1371/journal.ppat.1012646

**Published:** 2024-10-18

**Authors:** The PLOS Pathogens Editors

Concerns have been raised about the reliability of results reported in Figs 1, 4, 5, 7C, and S1, and S6 Figs of this article [1].

In response, the authors noted that some data underlying the figures of concern are no longer available. They also stated that errors in figure preparation may have led to some panel duplications, that published Fig 7C and S1 Fig include errors, that results of multiple experiments were pooled when preparing figures and analyzing results, and that not all replicates were included in some of the published quantitative results. They offered updated figures, original data (where available), and repeat data to address the issues.

A member of the journal’s Editorial Board advised that the responses and data provided by the authors did not resolve the issues. Therefore, the *PLOS Pathogens* Editors retract this article due to concerns about the reliability of the reported results and conclusions.

IC, PB, TV, GP, MG, and TL agreed with the retraction. DQ, JL, FB, AS, and RM either did not respond directly or could not be reached.
